# A brain MRI dataset and baseline evaluations for tumor recurrence prediction after Gamma Knife radiotherapy

**DOI:** 10.1038/s41597-023-02683-1

**Published:** 2023-11-08

**Authors:** Yibin Wang, William Neil Duggar, David Michael Caballero, Toms Vengaloor Thomas, Neha Adari, Eswara Kumar Mundra, Haifeng Wang

**Affiliations:** 1 Department of Industrial and Systems Engineering, Mississippi State University, Mississippi State, MS 39762 USA; 2 Department of Radiation Oncology, University of Mississippi Medical Center, Jackson, MS 39213 USA

**Keywords:** Radiotherapy, Computational models

## Abstract

Prediction and identification of tumor recurrence are critical for brain cancer treatment design and planning. Stereotactic radiation therapy delivered with Gamma Knife has been developed as one of the common treatment approaches combined with others by delivering radiation that targets accurately on the tumor while not affecting nearby healthy tissues. In this paper, we release a fully publicly available brain cancer MRI dataset and the companion Gamma Knife treatment planning and follow-up data for the purpose of tumor recurrence prediction. The dataset contains original patient MRI images, radiation therapy data, and clinical information. Lesion annotations are provided, and inclusive preprocessing steps have been specified to simplify the usage of this dataset. A baseline framework based on a convolutional neural network is proposed companionably with basic evaluations. The release of this dataset will contribute to the future development of automated brain tumor recurrence prediction algorithms and promote the clinical implementations associated with the computer vision field. The dataset is made publicly available on The Cancer Imaging Archive (TCIA) (10.7937/xb6d-py67).

## Background & Summary

A brain tumor is defined as a group of abnormal cells that grows in human brain tissues. Brain tumors are life-threatening by either directly invading and affecting healthy brain tissues or indirectly compressing other parts of the brain through tumor extension. Further damage will cause inflammation, brain swelling, and pressure within the skull. Brain tumors can be divided into two categories: benign and malignant. Benign tumors do not contain cancer cells and usually grow slowly. Malignant tumors which refer to brain cancer grow rapidly and invade healthy brain cells. In the year 2022, it is estimated that brain and other nervous system cancer has caused 18,280 deaths, and about 25,050 new cases were diagnosed^1^. A common type of brain tumor is brain metastases from other primary cancers such as breast, lung, and renal cancers. Clinicians may apply various therapies to treat these brain metastases. A detailed treatment plan will be discussed and made for each individual patient properly. Tumor cell size, location, and growth rate along with patient’s general health conditions will be considered. Current common treatment approaches include surgery, radiation therapy, chemotherapy, targeted therapy, hormone Therapy, or a combination of the above. Among those, stereotactic radiation therapy has been developed over the last few decades to deliver ionizing radiation that targets accurately via x-rays and *γ*-rays by linear accelerators or specialized devices such as the Gamma Knife while minimizing effects on nearby healthy tissues. Over half of cancer patients worldwide are treated with the radiation therapy approach or combined with surgery or chemotherapy approximately^2^.

In general, if the brain tumor is radiosensitive, clinicians may prescribe radiation therapy to treat cancer. Conventional radiation therapy aims for external beams of x-rays, *γ*-rays, or protons at the tumor to kill cancer cells and shrink brain tumors. Patients usually receive treatment over a specific period of time. The Gamma Knife device is used for the delivery of stereotactic radiation therapy during which high doses of radiation are delivered in 1 to 5 treatment sessions. Following radiation therapy, each tumor may be controlled, i.e., shrink or stable, or progress, i.e., recur. It is critical to monitor, identify, and evaluate patients during follow-up to radiation therapy for potential tumor recurrence. If a tumor could be accurately predicted as high-rish for recurrence, the current treatment planning choices would be directly impacted (i.e. prescription dose regimen or planning goals). The goal that motivates the collection of this brain tumor magnetic resonance imaging (MRI) dataset is to allow various artificial intelligence algorithms to model and predict if each current tumor (lesion) will progress or not based on the current treatment planning, imaging, and patient clinical information.

In this work, we release the fully publicly available brain cancer MRI dataset for the purpose of tumor recurrence identification and prediction. The data collection includes original patient MRI images and radiation therapy (RT) data that consists of RTStructure and RTDose files in Digital Imaging and Communications in Medicine (DICOM) format. Patient’s clinical records are also provided including gender, age, primary diagnosis, and course information. Each lesion is annotated and confirmed by radiologists and radiation oncologists. In addition to the dataset, we also develop a novel multi-input deep learning framework with a 3D convolutional neural network (CNN) as the baseline algorithm to predict the possible recurrence of a lesion. Deep learning methods have shown great success in tumor pathology, including tumor diagnosis, subtyping, grading, staging, and prognostic prediction, as well as the identification of pathological features^3^. It is still challenging for our proposed baseline model to identify the recurrence scenario particularly. Nevertheless, the release of the baseline algorithm will facilitate the development and evolution of an automated tumor recurrence classification framework. More innovative algorithms are expected to be explored to achieve acceptable clinical performance. Notably, the interpretability and explainability of the forthcoming machine learning algorithms are necessary for future clinical practice. Further potential applications based on this dataset include radiation treatment planning, evaluating and refining treatment, and automated dose delivery planning.

## Methods

This dataset was collected retrospectively under IRB-approval (2017-0266) from a clinical database of patients treated for brain metastases with Gamma Knife radiation therapy at the University of Mississippi Medical Center. Due to the retrospective nature of the data collection, none of the patients were consented for data publication. However, an IRB waiver of consent was obtained due to the impracticability of obtaining patient consent (many patients were either lost to follow-up or had expired at the time of data collection). In preparation for Gamma Knife treatment, an MRI was acquired on a 1.5 T Siemens Magnetom scanner utilizing the scan parameters described in the literature including correction for geometric distortion^4^. The included MRI series are T1 MPRAGE with Gadolinium contrast and a voxel size of 1 × 1 × 1 mm which acts as the primary planning dataset for Gamma Knife treatment for brain metastase. The original axial plane dimension of the MRI volumes is 256 × 256 while the slice thickness varies individually. For each patient, the treatment planning DICOM data was collected including the MRI dataset with its accompanying RTStruct indicating the lesions that were targeted using a given MRI. The RTDose information provides how the dose was deposited for each respective lesion/target. The recurrence of a lesion is identified by post-treatment MRI scans (followed up every three months for the first year). The images are pushed to GammaPlan and fused with the original treatment planning scan. A tumor is evaluated to be within isodose lines and then classified as stable or decreasing in size. Sometimes, if changes are identified at the edge of isodose lines, then there may be treatment-related changes or progression of the disease. To decide, an MR spectroscopy and perfusion (blood flow or not) scan is performed, and 3 molecular markers (choline, lactate, and NAA) are measured on spectroscopy. If lactate levels are high, then it is radionecrosis. If choline or NAA are elevated, it is classified as recurrence.

A keyed spreadsheet also houses various other relevant clinical information mentioned above as well as how many treatment sessions were used to deliver the dose for a target represented in the RTDose as it may have been delivered in as few as 1, but maybe as many as 5 treatment sessions. Additionally, each patient may have undergone more than one treatment course as they developed new lesions and/or treated lesions recurred. Even though certain tumor types are now commonly distinguished by their molecular status and not simply their histological primary lesion, this information is included in the primary when available. Also, with the limited dataset, we are concerned about having too many features with not enough data, especially since this feature does not apply to every cancer and would be most useful perhaps regarding only a unique cancer type rather than a generalized brain metastasis model. The dose units are in Gray (Gy, AKA J/kg) which is typical in radiation therapy. The dose was calculated using the TMR10 algorithm in the GammaPlanÂ® (version 11) software which does not consider tissue heterogeneity in the calculation but is consistent with previous Gamma Knife treatment practice across many institutions. Regarding another feature of the administration of corticosteroids, it is patient-dependent, i.e., if diabetic, there will be no steroids; If not diabetic, 5 days of steroids with a tapering dose will be applied. If radionecrosis on f/u is found, steroids for 1 month with 2 mg BID will be started. If symptomatic doesn’t respond to steroids, bevacizumab will be prescribed. On the other hand, the next option will be LITT (Lasers Interstitial Thermal Therapy) or surgery.

Treatment was ultimately delivered on the Gamma Knife Icon® at the same institution. After collection, all DICOM data (MRI images, RTStruct, and RTDose) was fully anonymized removing all protected health information and treatment-related dates. The anonymization process included mapping them to identifiers that matched the clinical information in the database spreadsheet. At this point, no one can track any of the data given to an individual patient.

**Fig. 1:**
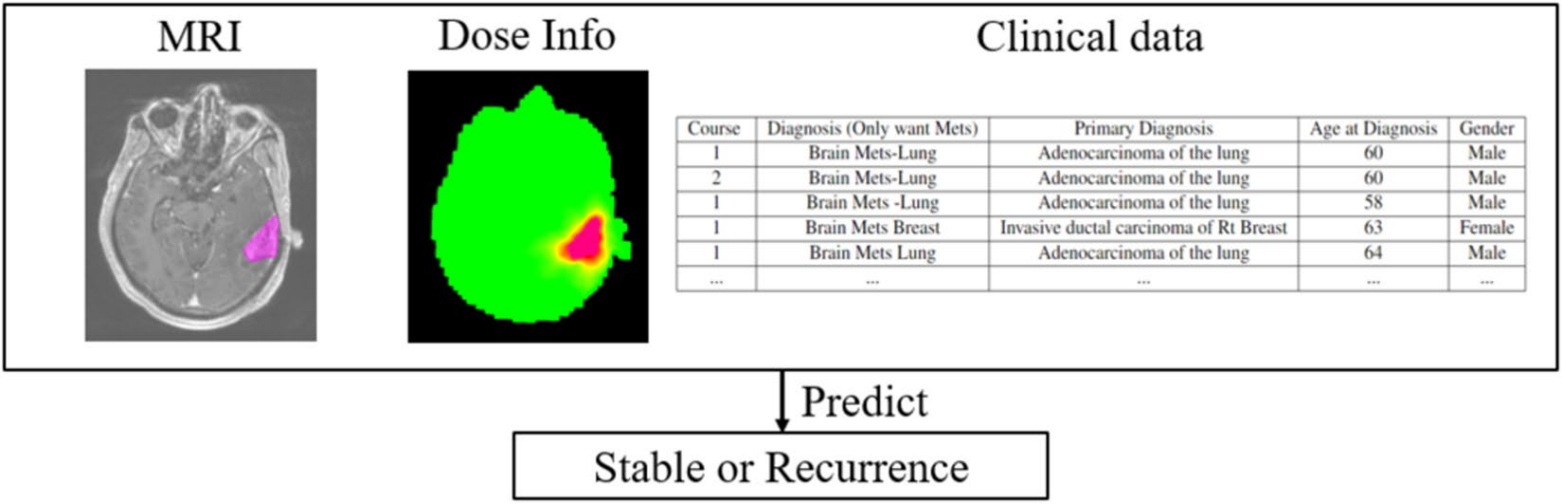
MRI dataset content and objective.

**Fig. 2:**
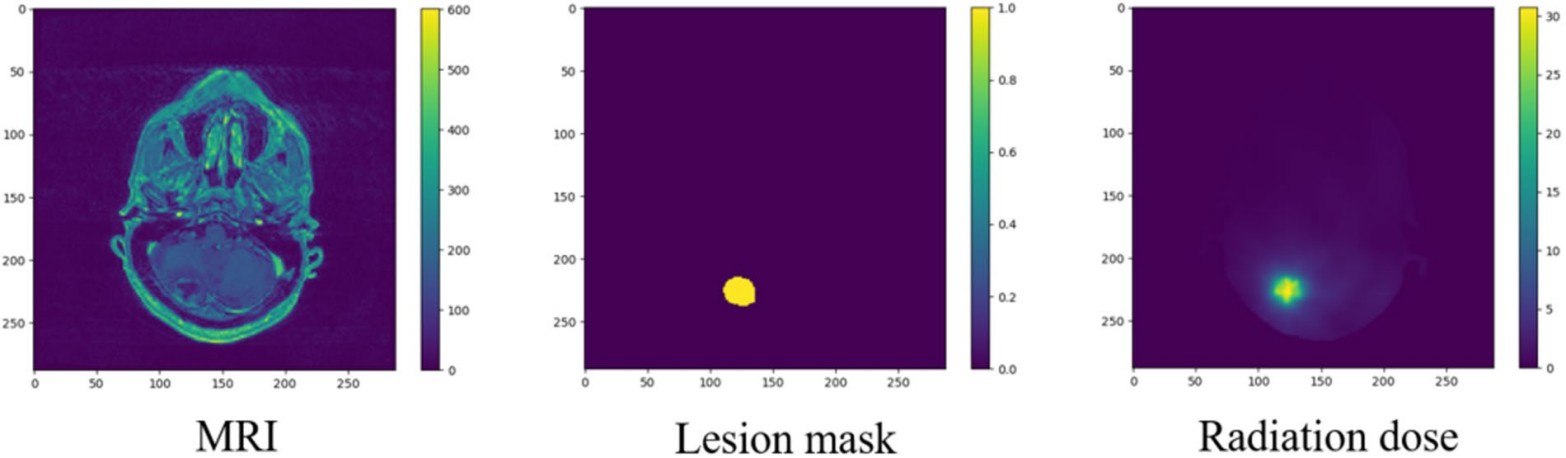
Sample patient MRI data with lesion annotation and dose radiation.

The aim of this data collection is to classify and predict brain tumor recurrence given a patient’s MRI, dose treatment plan, and clinical information. The general method is shown in Fig. 1. A classification framework to be developed should be multi-input by considering all three types of inputs. MRI along with the corresponding radiation dose can be analyzed as an image-like input. Therefore, various CNN frameworks are preferred for feature extraction or direct prediction. Numerical or categorical clinical variables can be converted to dense representations. Our proposed baseline model follows the typical 3D CNN workflow for identification and prediction. A sample of a patient’s brain MRI with the corresponding lesion mask and dose radiation information can be found in Fig. 2.

**Fig. 3:**
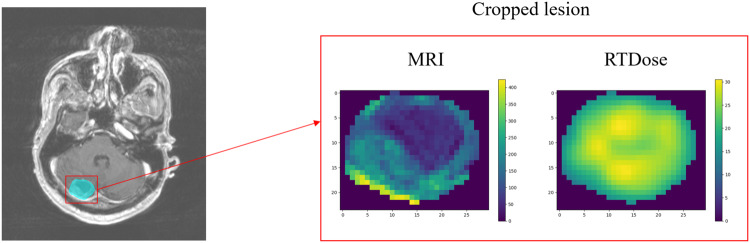
Studied lesion segmentation and extraction during data preprocessing.

We conduct a series of data preprocessing steps. First, dose MRI images have been resampled to the same spacing as the original patient MRI because the raw MRI and dose files share not only different dimensions but incompatible actual spacing as well. To represent the transformation process from the dose voxel *D* to MRI coordinates *M* by using affine transformation:

1 $$\left[\begin{array}{c}{M}_{x}\\ {M}_{y}\\ {M}_{z}\\ 1\end{array}\right]=\left[\begin{array}{cccc}{F}_{11}\Delta r & {F}_{12}\Delta c & {F}_{13}\Delta s & {D}_{x}\\ {F}_{21}\Delta r & {F}_{22}\Delta c & {F}_{23}\Delta s & {D}_{y}\\ {F}_{31}\Delta r & {F}_{32}\Delta c & {F}_{33}\Delta s & {D}_{z}\\ 0 & 0 & 0 & 1\end{array}\right]\left[\begin{array}{c}r\\ c\\ s\\ 1\end{array}\right]$$

where Δ*r*, Δ*c*, Δ*s* represent row, column, and slice voxel spacing resolution, respectively; *r*, *c*, *s* represent row, column, and slice index to their respective plane, and *F* refers to the values from each direction cosine of the image orientation. Thus, the dose voxel space can be further transformed to the corresponding original MRI voxel space. Second, every lesion region in each patient’s MRI has been extracted and cropped out based on the patient’s lesion mask. It is noted that one patient may have multiple lesions. Third, the corresponding radiation dose information can also be cropped out using the same lesion mask because the masks are shared through the same coordinate after resampling. In this way, each lesion MRI is paired with its radiation dose MRI. Furthermore, based on all the cropped lesions and dose MRI, the voxel values have been normalized between 0 and 1, respectively. The normalized common scale is demanded for the usage of machine learning algorithms. Resizing is necessary to keep the cropped training and test samples in the same dimension. The shape of 40 × 40 × 40 mm volume is determined, and further data augmentation techniques are discussed in the technical validation section. A sample procedure of lesion and dose extraction has been shown in Fig. 3.

## Data Records

The full dataset collection can be found on The Cancer Imaging Archive (TCIA) site^5^. In this data repository, we provide not only raw data but also preprocessed data. Raw DICOM files can be accessed and read by using open-source software, such as 3D Slicer (https://www.slicer.org/) or other software that focuses on image computing in clinical and biomedical fields. In general, this dataset represents patients who developed brain metastases for various primary tumors and then underwent Gamma Knife stereotactic radiation therapy in an effort to control the identified brain lesions. Additionally, these patients participated in treatment follow-up during which the response to treatment could be adequately assessed to identify recurring lesions and those that responded to treatment by stopping growth or even shrinking. For each patient, the treatment targets were identified and delineated through collaboration between radiation oncologists and neurosurgeons with validation from neuroradiologists in many cases.

**Fig. 4:**
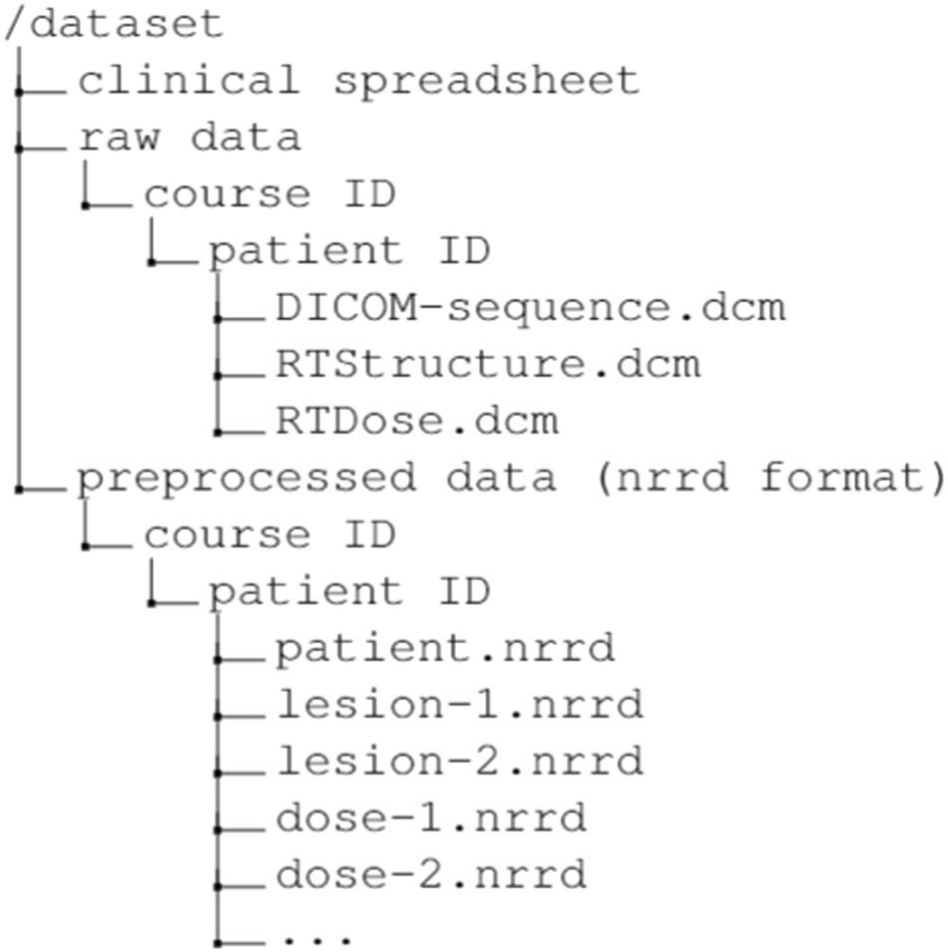
Data files and a directory structure of folders.

This public dataset retrospectively involved a total of 47 brain cancer patients with 21 males and 26 females. The dataset can be described in three ways: 1) patient-level, 2) course-level, and 3) lesion-level. Patient-level keeps all the data related to a specific patient altogether. Each course-level treatment data has been stored for each patient. Among all the patients, 17 patients received more than one course of treatment, 7 received more than two courses, and 1 patient received up to eight courses in total. Regarding the lesion-level, a total of 244 lesions were collected with annotations. 221 lesions are stable, and 23 are recurrence, which makes the dataset imbalance from the lesion-level perspective. Data files are organized through the flow of course-patient-lesion. The detailed data file directory structure is shown in Fig. 4. In terms of data format, we provide initial DICOM format MRI files for each patient. Lesion annotations and dose plan information are stored in RTStructure and RTDose, respectively. In addition to DICOM format, we provided Nearly Raw Raster Data (NRRD) format for neuroscience research support. NRRD is less complicated compared with DICOM while preserving essential metadata. Researchers can directly access those data formats without a start with processing RTStruct files. Additionally, all the preprocessed MRI images have been saved in numpy array format, which can facilitate the implementation in Python or other programming languages. Two sections of the data can be downloaded and studied separately. Different from the DICOM format usage where patient data consists of many slices, each patient MRI is retained in one single array along with the patient’s cropped lesion arrays. Corresponding dose information has also been extracted from RTStructure as a separate array file.

The clinical information has been collected in a spreadsheet that stores both patient-level and lesion-level records separately. Patient clinical data includes age, gender, age, course information, and primary diagnosis location. Lesion clinical data includes lesion property, location, and MRI type (stable or recurrence) associated with a specific patient. Clinical data samples of patient-level and lesion-level are shown in Tables 1, 2, respectively.

**Table 1:** Patient-level clinical data samples.

Unique patient ID	Course number	Diagnosis (Only want Mets)	Primary diagnosis	Age at diagnosis	Gender
463	1	Brain Mets-Lung	Adenocarcinoma of the lung	60	Male
463	2	Brain Mets-Lung	Adenocarcinoma of the lung	60	Male
247	1	Brain Mets Breast	Invasive ductal carcinoma of Rt Breast	63	Female
408	1	Brain Mets Lung	Adenocarcinoma of the lung	64	Male
…	…	…	…	…	…

**Table 2:** Lesion-level clinical data samples.

Unique patient ID	Course number	Lesion number	Lesion location	MRI type	Duration (months)
463	1	1	Lt Frontal	recurrence	11
463	2	2	R Motor Cortex	stable	8
463	2	3	Lt Post Temporal	stable	8
463	2	4	Lt Lat Cerebellum	stable	8
…	…	…	…	…	…

## Technical Validation

**Fig. 5:**
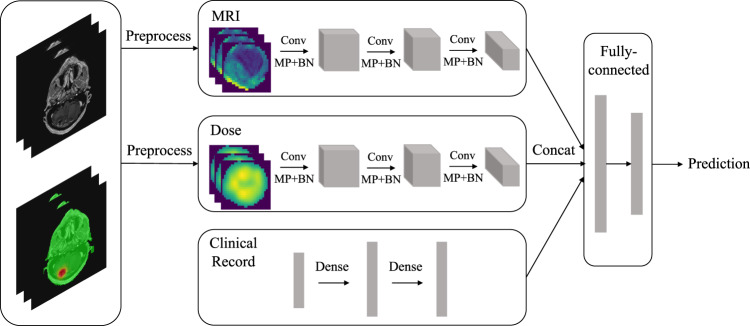
Overview of the proposed baseline network. (Clinical records: course, diagnosis, primary diagnosis, age at diagnosis, gender; Conv: convolution; MP: max pooling; BN: batch normalization; Concat: concatenate).

This dataset is validated and evaluated by our proposed baseline learning model. We apply a multi-input 3D CNN framework particularly, handling original patient MRI, dose history, and clinical information. A typical 3D convolution structure has been followed, including convolutional layers, pooling layers, and batch normalization. The neural network classifiers trained for volume inputs contain 3 × 3 × 3 convolutional layers with 32, 64, and 128 filters, respectively. Three types of inputs have been concatenated after convolution operations. Fully connected layers are used to generate the dense representation and the output category. An overview of the proposed baseline network is shown in Fig. 5. The total number of model parameters is 825,185. ADAM optimizer is chosen in our experiments. In terms of training and test data split, a set of test samples has been selected and reviewed by experts. In our baseline study evaluation, a random data split was performed on the subject level instead of the lesion level. Therefore, test performance can avoid being inflated as a lesion evaluated would not be included in the training for the same patient. Other split strategies may be investigated. Table 3 shows the lesion-level training and test set statistics. Recurrence lesions are rare in clinical practice, which results in a difference in terms of the number of samples in each category.

**Table 3:** Training and test set size before data augmentation.

Lesions	Stable	Recurrence	Total
Training Set	140	13	153
Test Set	81	10	91
Total	221	23	244

Since the dataset is imbalanced in terms of lesion-level, data augmentation techniques have been applied to train a more generalized machine learning model. Specifically, rotation operation is employed. Because an MRI image can be viewed as a 3D object, one single MRI can be rotated in three directions. Each object has been rotated with three angles, [90, 180, 270]. Dose MRI images have also been rotated and paired with patient MRI. Therefore, one sample can be augmented to nine more samples for training. The size of the augmented dataset is shown in Table 4.

**Table 4:** Training and test set size after data augmentation.

Lesions	Stable	Recurrence	Total
Training Set	140	130	270
Test Set	81	100	181
Total	221	230	451

The lesion recurrence identification performance is shown in Table 5. The model has achieved 90.1% accuracy, 10% sensitivity, 89.0% specificity, and 18.2% F1-score. Various cutting-edge techniques such as class weights remain open for further study to improve the performance due to the imbalanced dataset. According to the classification confusion matrix, it is demonstrated that the proposed baseline model is able to identify stable tumors accurately. However, lesion recurrence remains a challenging and open problem. Further potential improvement in model structure and important features of recurrence tumors are expected to be studied.

**Table 5:** Confusion matrix of the proposed baseline framework.

Lesion(s)	Predicted Stable	Predicted Recurrence	Total
Actual Stable	81	0	91
Actual Recurrence	9	1	10
Total	90	1	91

## Usage Notes

The full dataset can be found in the TCIA^5^. The data described in this work corresponds to the version reviewed in August 2023. Future updates can be referred within the same collection. Preprocessed data has been generated based on the approaches mentioned in the Methods section. Particularly, basic downstream processing steps such as resampling, VOI extraction, normalization, and resizing have been used. SimpleITK (https://simpleitk.org/) and Pydicom (https://pydicom.github.io/) packages are used in preprocessing. Users may have other preprocessing procedures in terms of different focus and implementation. We provide the script of our proposed baseline model based on the TensorFlow platform. Other advanced frameworks and optimization techniques are expected to be developed, so as to achieve promising results and promote the implementation of artificial intelligence-assist tumor recurrence prediction.

This dataset is published under a data use agreement between UMMC and the University of Arkansas for Medical Sciences (UAMS) that operates the TCIA. The agreement sets forth the terms by which UAMS will facilitate the participation in submission activities for TCIA research and publication programs. Users can access imaging data under a standard TCIA Data Use Agreement. Clinical data is accessible under a CC-BY license. A project description must be provided by the user. Some data in this collection contains images that could potentially be used to reconstruct a human face. To safeguard the privacy of participants, users must sign and submit a restricted license agreement to TCIA before accessing the data. Users who use the dataset should properly acknowledge the data contributions of the authors by citing this article and the data repository. It is noted that lesion segmentation can be subjective and manual errors may occur. Any issues or feedback can be sent to the authors, and any unexpected errors can be posted on the data repository page. We will also keep the repository updated when we release any new data.

## Data Availability

The repository of brain tumor recurrence prediction data can be found on our GitHub (https://github.com/siolmsstate/brain_mri). Pydicom version 2.3.0 and SimpleITK version 2.1.0 have been used in data preprocessing. The baseline model framework is generated using TensorFlow version 2.8.0. We release sample codes for users to get started with raw data, guiding through loading the data and all preprocessing steps. Fundamental data visualization is also available.
